# Sand fly fauna of Crete and the description of *Phlebotomus* (*Adlerius*) *creticus* n. sp. (Diptera: Psychodidae)

**DOI:** 10.1186/s13071-020-04358-x

**Published:** 2020-11-04

**Authors:** Vít Dvořák, Nikolaos Tsirigotakis, Christoforos Pavlou, Emmanouil Dokianakis, Mohammad Akhoundi, Petr Halada, Petr Volf, Jérôme Depaquit, Maria Antoniou

**Affiliations:** 1 Department of Parasitology, Faculty of Science, Charles University, Prague, Czech Republic; 2 Laboratory of Clinical Bacteriology Parasitology Zoonoses and Geographical Medicine, Faculty of Medicine, University of Crete, Heraklion, Greece; 3 Département de Parasitologie-Mycologie, Hôpital Avicenne AP-HP, Bobigny, France; 4 BioCeV – Institute of Microbiology, The Czech Academy of Sciences, Vestec, Czech Republic; 5 FEA7510 “ESCAPE”, USC ANSES “VECPAR”, Faculté de Pharmacie, Université de Reims Champagne-Ardenne, 51, rue Cognacq-Jay, 51096 Reims cedex, Reims, France

**Keywords:** Phlebotominae, *Phlebotomus* (*Adlerius*) *creticus* n. sp., Crete, Greece, Sand fly fauna

## Abstract

**Background:**

The Greek island of Crete is endemic for both visceral leishmaniasis (VL) and recently increasing cutaneous leishmaniasis (CL). This study summarizes published data on the sand fly fauna of Crete, the results of new sand fly samplings and the description of a new sand fly species.

**Methods:**

All published and recent samplings were carried out using CDC light traps, sticky traps or mouth aspirators. The specific status of *Phlebotomus* (*Adlerius*) *creticus* n. sp., was assessed by morphological analysis, *cytochrome b* (*cytb*) sequencing and MALDI-TOF protein profiling.

**Results:**

Published data revealed the presence of 10 *Phlebotomus* spp. and 2 *Sergentomyia* spp. During presented field work, 608 specimens of 8 species of *Phlebotomus* and one species of *Sergentomyia* were collected. Both published data and present samplings revealed that the two most common and abundant species were *Phlebotomus neglectus*, a proven vector of *Leishmania infantum* causing VL, and *Ph. similis*, a suspected vector of *L. tropica* causing CL. In addition, the field surveys revealed the presence of a new species, *Ph.* (*Adlerius*) *creticus* n. sp.

**Conclusions:**

The identification of the newly described species is based on both molecular and morphological criteria, showing distinct characters of the male genitalia that differentiate it from related species of the subgenus *Adlerius* as well as species-specific sequence of *cytb* and protein spectra generated by MALDI-TOF mass spectrometry. 
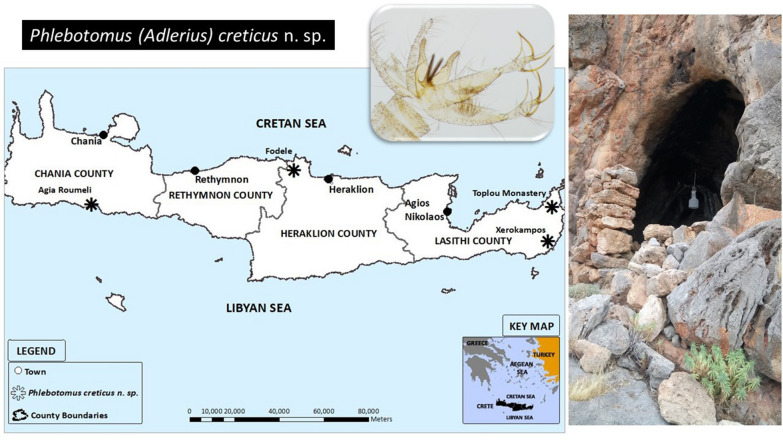

## Background

Phlebotomine sand flies (Diptera: Psychodidae) are hematophagous insects that transmit the protozoan parasites *Leishmania* spp. as well as the bacterium *Bartonella bacilliformis* and viruses (phleboviruses, vesiculoviruses and orbiviruses) [1]. Four medically important *Leishmania* species circulate in various regions of the Mediterranean basin: *L. infantum* causing both zoonotic visceral (VL) and anthroponotic cutaneous (CL) leishmaniasis in humans and canine leishmaniasis in dogs (CanL); *L. major* causing zoonotic CL; *L. tropica* causing anthroponotic or zoonotic CL [2–4] and the recently introduced *L. donovani* causing both anthroponotic VL and CL [5].

Crete is endemic for VL and CanL caused by *L. infantum*, with an increasing number of CL every year caused by *L. tropica* whilst the danger of the introduction of new species/zymodemes is enhanced by the arrival of refugees from endemic areas [6]. The key factor for determining the distribution and spread of leishmaniases is by recording the geographical distribution and abundance of the medically important sand fly vectors. Generally, in Greece, proven or suspected vectors are *Ph. similis* Artemiev & Neronov, 1984 for *Leishmania tropica* and *Ph. perfiliewi* Parrot, 1930, *Ph. tobbi* Adler & Theodor, 1930 and *Ph. neglectus* Tonnoir, 1921 for *L. infantum* [7].

The main aims of this study were to review historical data on the sand fly fauna of Crete and describe a new *Phlebotomus* (*Adlerius*) species sampled during surveys carried out from 2014 to 2019. To confirm the status of the new species, in addition to morphological and genetic criteria, for the first time, protein profiling using MALDI-TOF mass spectrometry was also deployed.

## Methods

### Sand fly data

All published data recording sand fly presence in Crete were gathered. The literature search concerning the review follows the Prisma Journal Publishing protocol workflow [8]. PubMed, Web of Science, Google Scholar databases and web searches were screened from 1910 to 30 November 2019, using the following keywords: Phlebotominae, *Phlebotomus*, Crete, *Sergentomyia*, Greece, sand flies, CDC light traps, sticky traps, mouth aspirator, electric aspirator. Full text articles in English containing information on phlebotomine sand flies from Crete were selected. Other articles, including those in other languages, that contain desired information were also included, based on the cited databases knowledge of the authors. Samplings were carried out using CDC miniature light traps (John W. Hock Co., Gainesville, FL, USA), Sticky Traps (A4 paper coated with castor oil) or mouth aspirators. The sampling sites were mostly animal farms, houses, schools, churches, deserted houses, wells, caves and rural areas with different cultivations or wild vegetation.

### Study areas and sampling

The new samplings were carried out in five study areas, two in Heraklion, two in Chania and one in Lasithi between 2014 and 2019 (Table 1). The study areas were Fodele and Foinikia in Heraklion prefecture, Agia Roumeli and Botanical Gardens in Chania and Xerokampos in Lasithi. The CDC miniature light traps equipped with a fine net cage were used for all the samplings. In all sampling areas, 7–9 light traps were placed in different microhabitats for one to two days. The light traps were set 1.5 h before sunset and were collected 2 h after dawn. Specifically, in Fodele 9 light traps for 2 sampling nights in May 2019 were used, in Foinikia 9 traps for 1 night in August of 2018, in Agia Roumeli 7 traps for 2 sampling nights in May of 2014, and in Botanical Garden 9 traps for 2 sampling nights in August of 2019. In Xerokampos two samplings were carried out during April of 2014 and in May of 2019 using 9 traps for 2 sampling nights.

**Table 1:** The study areas of the 2014–2019 samplings in Crete

Study area	Latitude	Longitude	Date	Trap locations
Xerokampos	35° 3′ 29.37′′ N	26° 14′ 27.14′′ E	14–16 April 2014	Caves
Agia Roumeli	35° 13′ 24.77′′ N	23° 56′ 7.53′′ E	11–12 May 2014	Caves
Foinikia	35° 16′ 31.73′′ N	25° 6′ 16.48′′ E	28 August 2018	Dogs, olive trees
Fodele	35° 22′ 53.93′′ N	24° 57′ 28.27′′ E	24–26 May 2019	Hencoop, trees, small cave
Xerokampos	35° 3′ 29.37′′ N	26° 14′ 27.14′′ E	28–30 May 2019	Caves
Botanical Garden	35° 25′ 6.69′′ N	23° 56′ 23.08′′ E	2–3 August 2019	Animals, trees

### Sand fly morphological identification

The specimens were stored in vials containing absolute ethanol HPLC grade (Thermo Fischer Scientific, Gloucester, UK) at room temperature prior to mounting, except for the specimens later analyzed by MALDI-TOF protein profiling which were stored in 70% molecular grade ethanol at − 20 °C before further processing. The samples were divided into specimens mounted *in toto* and specimens processed for molecular biology. In the latter case, the head, thorax including wings and genitalia were removed and placed in a drop of ethanol before their processing similar to specimens mounted *in toto*, while the other parts of the body were kept in ethanol for molecular analysis. Soft tissues were lysed in a bath of KOH 10%, then bleached in Marc-André solution, and mounted between microscope slide and cover slide in Euparal^®^ for species identification after dehydration in graded ethanol series [9]. Some specimens were mounted immediately after clearing in Marc-André solution or in high viscosity CMCP-10 medium (Polysciences, Inc., Warrington, PA, USA). Morphological identification was performed under a BX61 microscope (Olympus, Japan). Measurements and counts were taken using the Stream Motion software (Olympus, Japan) and a video camera connected to the microscope. Identification was performed based on the keys available for Crete and adjacent regions [10–14].

**Fig. 1:**
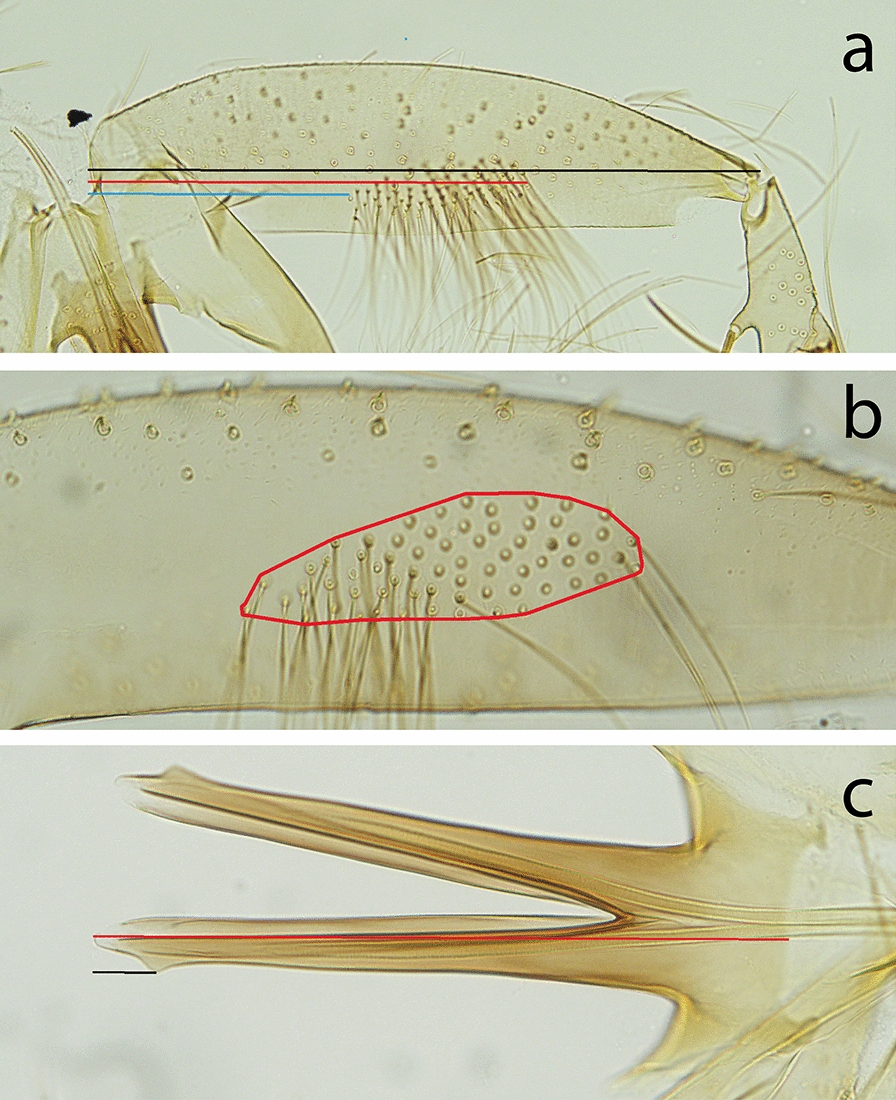
Measurements of male genitalia. **a** Measurements of the length of the gonocoxite (in black), beginning of the internal tuft of setae (in blue) and ending of the internal tuft of setae (in red). **b** Area of the internal tuft of setae of the gonocoxite. **c** Parameral sheath length (in red) and distance from the tubercle to the top of the parameral sheath (in black)

The terminology adopted for the characters is the most recent one for phlebotomine sand flies [15]. The following measurements were made for the specimens of the new species for both sexes: flagellomeres 1, 2 and 3, labrum-epipharynx. For males, we also measured the lengths of the parameral sheath, the distance from the tubercle to the tip of the parameral sheath (indicated as the distance from the tip of the aedeagus to the subterminal tooth by Artemiev [10]), sperm pump, aedeagal ducts, gonocoxite, beginning and ending of the tuft of internal setae of the gonocoxite, number of internal setae of the gonocoxal tuft and area of the internal tuft of setae of the gonocoxite (Fig. 1).

The percentage of tuft length *vs* gonocoxite length was calculated as follows: tuft length × 100)/gonocoxite length. The median tuft position *vs* gonocoxite was calculated as follows: beginning of the tuft of internal setae of the gonocoxite + tuft length/2) × 100/gonocoxite length. Drawings were made using a camera lucida.

### DNA extraction and *cytochrome b* mtDNA sequencing

Following morphological identification, DNA was extracted using DNeasy Blood & Tissue Kit (Qiagen, Hilden, Germany) from 16 individuals (males and females) of *Ph.* (*Adlerius*) *creticus* n. sp. collected during previous surveys at four localities in Crete as well as from two specimens of *Ph. simici* Nitzuescu, 1931 from Crete and three specimens of *Ph. balcanicus* Theodor, 1958 from Iran. *cytb* PCR assays were performed using primers and conditions described by Esseghir et al. [16] and sequenced both directions according to Sanger’s method using the same primers as in the PCR assay.

### Alignment and genetic distances

The newly generated sequences data were aligned with *cytb* sequences available on GenBank (Table 2), which belong to *Adlerius* spp. [17–21]. Multiple sequence alignments were performed using ClustalW [22] as implemented in MEGA7 [23]. Genetic distances were calculated between and within species using Tamura-Nei model [24], also in MEGA7 [23].

**Table 2:** Information on specimens used for *cytb* sequencing analysis

Sample	Geographical origin	Collection date	GenBank ID
Crete12	Crete, Xerokampos	30 May 2019	MT501623
Crete13	Crete, Xerokampos	30 May 2019	MT501624
Crete14	Crete, Xerokampos	30 May 2019	MT501625
Crete17	Crete, Xerokampos	30 May 2019	MT501626
Crete18	Crete, Xerokampos	30 May 2019	MT501627
Crete19	Crete, Xerokampos	30 May 2019	MT501628
AR112	Crete, Agia Roumeli	11 May 2014	MT501629
AR224	Crete, Agia Roumeli	11 May 2014	MT501630
AR231	Crete, Agia Roumeli	11 May 2014	MT501631
F235	Crete, Fodele	2013	MT501632
Fb16	Crete, Fodele	2013	MT501633
Fb19	Crete, Fodele	2013	MT501634
BAGR2	Crete, Toplou monastery	11 August 1991	MT501635
BAGR7	Crete, Toplou monastery	11 August 1991	MT501636
BAGR4	Crete, Toplou monastery	11 August 1991	MT501637
BAGR8	Crete, Toplou monastery	11 August 1991	MT501638
BAIR4	Iran, Kaleybar	July 2010	MT501639
BAIR5	Iran, Kaleybar	July 2010	MT501640
BAIR100	Iran, Kaleybar	July 2010	MT552617
SICR2	Crete, Messa Mouliana	13 August 1991	MT552618
SICR3	Crete, Messa Mouliana	13 August 1991	MT552619
*Ph. brevis*	Iran	1985	HQ023282
*Ph. turanicus*	Afghanistan	2009	HM803195
*Ph. halepensis*	Iran	2006–2007	HQ391905
*Ph. halepensis*	Iran	2005	HQ023283
*Ph. chinensis*	China	na	HM747274
*Ph. chinensis*	China	na	HM747272
*Ph. chinensis*	China	na	HM747268
*Ph. chinensis*	China	na	HM747264
*Ph. chinensis*	China	na	HM747260
*Ph. perfiliewi*	Italy	na	KF680811

na, not available

### Phylogenetic analyses

The optimal nucleotide substitution models were identified using PartitionFinder (PF) v.2.1.1 [25]. We ran PF two different times using the greedy search algorithm with linked branch lengths in calculations of likelihood scores under the Bayesian information criterion (BIC). The difference between these two runs was the restriction of candidate models to only those that are available in either MrBayes v.3.2.6 [26] or PhyML v.3.0 [27]. The models which included both gamma distribution (G) and invariable sites (I) were ignored [28].

Phylogenetic trees were constructed using Bayesian Inference (BI) and Maximum Likelihood (ML) methods. The BI analysis was performed in MrBayes v3.2.6 [26] with 4 runs and 8 chains per run for 10,000,000 generations, with a sampling frequency of 100. From the sampled trees, 25% were discarded as ‛burn-inʼ phase and therefore, a majority rule consensus tree relied on the remaining trees and posterior probabilities were calculated as the percentage of samples recovering any particular clade [29]. ML analysis was performed with PhyML v.3.0 [27] with nearest-neighbor-interchange search, bio-neighbor joining starting tree under the suggested models selected in PF. Bootstrap values were estimated by 1000 replicates [30]. *Phlebotomus perfiliewi* represented the outgroup in the phylogenetic analyses.

### MALDI-TOF protein profiling

Samples that were subjected to MALDI-TOF MS analysis were processed as previously described [31]. It was demonstrated that mass spectrometry-based approach is not suitable for specimens collected by sticky traps [32]; therefore, only specimens collected by CDC light traps were included into the assay. Within one month after the collection in the field, specimens stored in 70% ethanol were dissected, heads and terminalia were mounted by CMCP-10 mounting medium (Polysciences) for morphological typing, rest of abdomens were stored for DNA isolation and *cytb* sequencing as described above and thoraxes were ground by a manual BioVortexer homogenizer (BioSpec, Bartlesville, USA) with sterile disposable pestles in 10 μl of 25% formic acid. Two µl of the homogenate were mixed with 2 µl of freshly prepared MALDI matrix, an aqueous 60% acetonitrile/0.3% TFA solution of sinapinic acid (30 mg/ml; Sigma-Aldrich, St. Louis, USA). One μl of this mixture were spotted directly onto a steel MALDI plate in duplicates. Protein mass spectra were measured using an Autoflex Speed MALDI-TOF spectrometer (Bruker Daltonics, Billerica, USA) in a mass range of 4–25 kDa and compared by FlexAnalysis 3.4 software. For cluster analysis and species identification, the protein profiles were processed using MALDI Biotyper 3.1 and searched against an in-house database that comprises reference spectra of 23 different sand fly species including following *Adlerius* species: *Ph. arabicus* Theodor, 1953, *Ph. balcanicus*, *Ph. halepensis* Theodor, 1958 and *Ph. simici*.

## Results

### Sand fly data

The first published study of the sand fly fauna of Crete appeared in 1910 and since then, 16 more works were published, revealing the presence of 10 *Phlebotomus* spp. and 2 *Sergentomyia* spp. (Table 3).

**Table 3:** Publications on the sand fly fauna of Crete [34–50]

Species	Reference	Prefecture
*Ph.* (*Larroussius*) *neglectus* Tonnoir, 1921	Parrot [34]; Adler et al. [35]; Ristorcelli [36]; Hertig [37]; Hadjinicolaou [38]; Pesson et al. [39]; Léger et al. [40]; Chaniotis et al. [41]; Ivović et al. [42]; Ivović et al. [43]; Christodoulou et al. [44]; Alten et al. [45]	Chania, Rethymno, Heraklion, Lasithi
*Ph.* (*Lar.*) *tobbi* Adler & Theodor, 1930	Langeron [46]; Ivović et al. [42]; Christodoulou et al. [44]	Chania, Heraklion
*Ph.* (*Lar.*) *perfiliewi* Parrot, 1930	Pesson et al. [39]; Léger et al. [40]; Christodoulou et al. [44]	Rethymno, Heraklion, Lasithi
*Ph.* (*Adlerius*) *simici* Nitzulescu, 1931	Parrot [34]; Adler et al. [35]; Ristorcelli [36]; Hertig [37]; Hadjinicolaou [38]; Léger et al. [40]; Aransay et al. [47]; Christodoulou et al. [44]	Chania, Rethymno, Heraklion, Lasithi
*Phlebotomus.* (*Adlerius*) sp.	Léger et al. [40]; Christodoulou et al. [44]	Chania, Rethymno, Heraklion
*Ph.* (*Phlebotomus*) *papatasi* Scopoli, 1786	Birt [50]; Blanc and Caminopetros [48]; Langeron [46]; Parrot [34]; Adler et al. [35]; Ristorcelli [36]; Hertig [37]; Hadjinicolaou [38]; Léger et al. [40]; Aransay et al. [47]; Ivović et al. [42]; Christodoulou et al. [44]; Alten et al. [45]	Chania, Rethymno, Heraklion, Lasithi
*Ph.* (*Paraphlebotomus*) *alexandri* Sinton, 1928	Aransay et al. [47]; Christodoulou et al. [44]	Chania, Heraklion
*Ph.* (*Par.*) *similis* Artemiev & Neronov, 1984	Blanc & Caminopetros [48]; Langeron [46]; Parrot [34]; Adler et al. [35]; Ristorcelli [36]; Hadjinicolaou [38]; Léger et al. [40]; Aransay et al. [47]; Ivović et al. [42]; Christodoulou et al. [44]; Alten et al. [45]	Chania, Rethymno, Heraklion, Lasithi
*Ph.* (*Transphlebotomus*) *mascittii* Grassi, 1908	Parrot [34]; Adler et al. [35]; Ivović et al. [42]; Christodoulou et al. [44]	Chania, Heraklion, Lasithi
*Ph.* (*Tra.*) *killicki* Dvorak, Votypka & Volf, 2015	Kasap et al. [49]	Chania
*Se.* (*Sergentomyia*) *minuta* Rondani, 1843	Langeron [46]; Parrot [34]; Adler et al. [35]; Hertig [37]; Hadjinicolaou [38]; Léger et al. [40]; Aransay et al. [47]; Ivović et al. [42]; Alten et al. [45]	Chania, Rethymno, Heraklion, Lasithi
*Se.* (*Ser.*) *dentata* Sinton, 1933	Ivović et al. [42]	Heraklion
*Sergentomyia* sp.	Christodoulou et al. [44]	Chania, Rethymno, Heraklion, Lasithi

### Sand fly sampling in 2014–2019

Overall, 608 sand fly specimens were collected which corresponded to nine different species with the most common and abundant species being *Se. minuta* and *Ph. neglectus* (Table 4). In addition to species known in Crete, 151 specimens of *Ph.* (*Adlerius*), morphologically close to *Ph. balcanicus* and *Ph. zulfagarensis* were identified. These specimens were further analysed and their morphological and molecular identification is described below.

**Table 4:** Sand fly species found in the 2014 to 2019 samplings in Heraklion (Foinikia, Fodele), Lasithi (Xerokampos) and Chania (Agia Roumeli, Botanical Garden)

Species	Foinikia (2018)	Fodele (2019)	Xerokampos (2014)	Xerokampos (2019)	Agia Roumeli (2014)	Botanical Garden (2019)	Total
*Ph. neglectus*	2	61	8	101	7	42	221
*Ph. tobbi*	0	0	0	1	0	0	1
*Ph. simici*	0	0	0	4	0	25	29
*Ph. creticus* n. sp.	0	0	15	131	5	0	151
*Ph. papatasi*	1	0	0	0	0	0	1
*Ph. similis*	1	0	0	29	3	0	33
*Ph. alexandri*	0	0	0	0	0	30	30
*Ph. killicki*	0	0	0	1	0	4	5
*Se. minuta*	29	0	0	32	0	76	137
Total	33	61	23	299	15	177	608

**Fig. 2:**
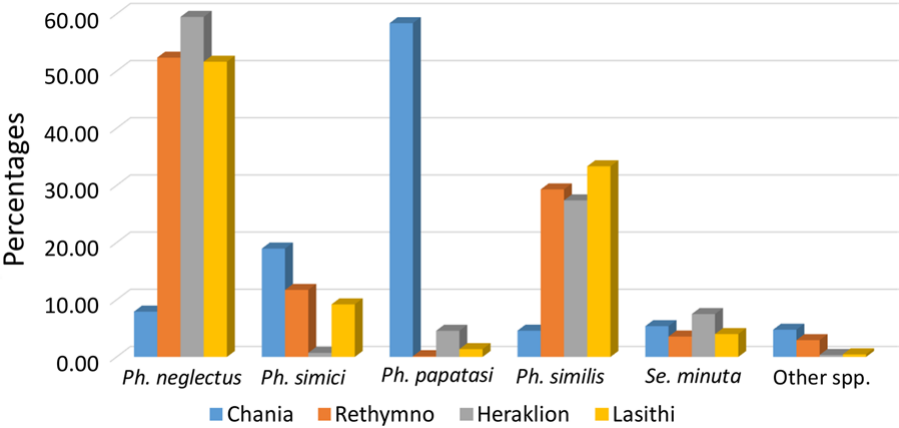
Percentages of species collected per prefecture in both published literature and present samplings

Table 5 sums up the total number of specimens, of each species, caught in present samplings and previous publications. More than 30,000 specimens have been reported and about 63.27% of them were identified as the medically important species *Ph. neglectus*. Moreover, a significant proportion of the specimens were identified as *Ph. similis* (17.4%) and *Ph. papatasi* (9.49%). In all prefectures except Chania, *Ph. neglectus* (> 50%) and *Ph. similis* (~30%) were the most common and abundant species. In Chania, *Ph. papatasi* was the most common and abundant species which comprised almost the 60% of the sand fly specimens (Fig. 2).

**Table 5:** Estimated total numbers of sand fly species reported both in publications and present samplings in Crete

Species	No. of specimens	Percentage
*Ph. neglectus*	~ 20,000	~ 63.27
*Ph. tobbi*	6	~ 0.02
*Ph. perfiliewi*	5	~ 0.02
*Ph. simici*	~ 1300	~ 4.11
*Phlebotomus* (*Adlerius*) sp.	~ 35	~ 0.11
*Ph. creticus* n. sp.	151	~ 0.48
*Ph. papatasi*	~ 3000	~ 9.49
*Ph. alexandri*	~ 70	~ 0.22
*Ph. similis*	~ 5500	~ 17.40
*Ph. mascittii*	30	~ 0.09
*Ph. killicki*	18	~ 0.06
*Se. minuta*	~ 1000	~ 3.16
*Se. dentata*	21	~ 0.07
*Sergentomyia* sp.	475	~ 1.50

### Morphological typing of *Ph. creticus* n. sp

Fifty-four male individuals collected by CDC light traps were used for the morphological analysis, originating from three localities (8 from Toplou, 23 from Xerokampos and 23 from Fodele). The descriptive statistics for 22 characters are given in Table 6. Eleven females from Xerokampos were used for the description, descriptive statistics for 6 characters are given in Table 7. To exclude a presence of two distinct populations, the normality of the distributions of each of the morphological characters was tested by Shapiro-Wilk test (*P* > 0.05) and was found not to be significantly different from normal.

**Table 6:** Descriptive statistics for the measurements and counts for males of *Ph. creticus* n. sp.

Character^a^	No. of specimens	Range	Mean	SD
Flagellomere 1 (A III)	45	285–406	340	28.13
Flagellomere 2 (A IV)	45	132–173	150.8	11.52
Flagellomere 3 (A V)	44	127–168	148.4	10.47
Flagellomere 1/flagellomere 2 + flagellomere 3	44	1.01–1.25	1.13	0.05
Labrum	50	237–310	264.2	15.4
Flagellomere 1/labrum	45	1.14–1.53	1.29	0.09
Parameral sheath length	54	178–219	195.3	10.84
Distance between the tubercle and the top of the parameral sheath	54	8–21	15.3	2.49
Gonocoxite length	53	345–451	405.2	22.28
Beginning of the tuft of internal setae of the gonocoxite	53	100–191	148.4	17.7
% beginning *vs* gonocoxite length	53	29–43	36.5	2.85
Ending of the tuft of internal setae of the gonocoxite	53	212–305	260.3	19.01
% ending *vs* gonocoxite length	53	60–68	64.2	1.91
Tuft length	53	92–129	111.9	8.36
% tuft length *vs* gonocoxite length	53	22–33	27.7	2.26
Median tuft position *vs* gonocoxite	53	45–55	50.4	2.15
Sperm pump	26	119–157	133.2	9.61
Aedeagal ducts	31	725–1082	935.6	86.07
Aedeagal ducts/sperm pump	26	6–8	6.95	0.66
Number of setae	50	54–85	69.3	6.69
Gonocoxal internal setae area (µ^2^)	43	2553–4360	3465	460.78
Mean µ^2^ per seta of the gonocoxal internal tuft	43	40–64	50.4	4.61

SD, standard deviation

^a^ Measurements in µm

**Table 7:** Descriptive statistics for the measurements and counts for females of *Ph. creticus* n. sp.

Character^a^	No. of specimens	Range	Mean	SD
Flagellomere 1 (A III)	11	268–326	298	16.92
Flagellomere 2 (A IV)	11	113–140	125.45	7.96
Flagellomere 3 (A V)	11	108–141	125.37	8.62
Flagellomere 1/flagellomere 2 + flagellomere 3	11	316–372	338.87	17.20
Labrum	11	1.13–1.27	1.19	0.04
Flagellomere 1/labrum	11	0.80–0.94	0.88	0.05

SD, standard deviation

^a^ Measurements in µm

The antennal formula in males shows variability. Of the 44 males examined exhibiting antennae, 36 have an antennal formula 2/f1-f3, 1/f4-f13 which appears as the most common formula. Five specimens exhibit the following formula: 2/f1-f4, 1/f5-f13 with very commonly one very small ascoid on f5. One specimen exhibits 2/f1-f2, 1/f3-f13. One specimen exhibits 2/f1-f5, 1/f6-f13 with a small ascoid on both f4 and f5. Interestingly, one specimen exhibits 2/f1-f4, 1/f5-f13 on the left antenna and 2/f1-f3 + f5, 1/f4 + f6-f13 on the right one.

### Sequence analysis

The *cytb* gene was successfully amplified and sequenced and the final dataset consisted of an alignment of 446 bp. The pairwise distances between species ranged from 0.044 to 0.188, while the closest related species to *Ph. creticus* n. sp. appeared to be *Ph. balcanicus* with a mean distance of 0.044. The mean distance within the *Ph. creticus* n. sp. samples was 0.008. The pairwise mean distances between and within species are provided in Table 8.

**Table 8:** *cytb* sequence distances between and within (values in italic) the species analyzed under the Tamura-Nei model

Species		1	2	3	4	5	6	7	8
1	*Ph. creticus* n. sp.	*0.008*							
2	*Ph. balcanicus*	0.044	*0.006*						
3	*Ph. simici*	0.111	0.107	*0.002*					
4	*Ph. brevis*	0.137	0.115	0.064	*–*^*a*^				
5	*Ph. turanicus*	0.097	0.089	0.108	0.112	*–*^*a*^			
6	*Ph. halepensis*	0.109	0.077	0.140	0.113	0.103	*0.011*		
7	*Ph. chinensis*	0.124	0.128	0.130	0.134	0.138	0.168	*0.011*	
8	*Ph. perfiliewi*	0.129	0.137	0.152	0.182	0.141	0.188	0.159	*–*^*a*^

^a^Only one specimen sequenced

### Phylogenetic analyses

**Fig. 3:**
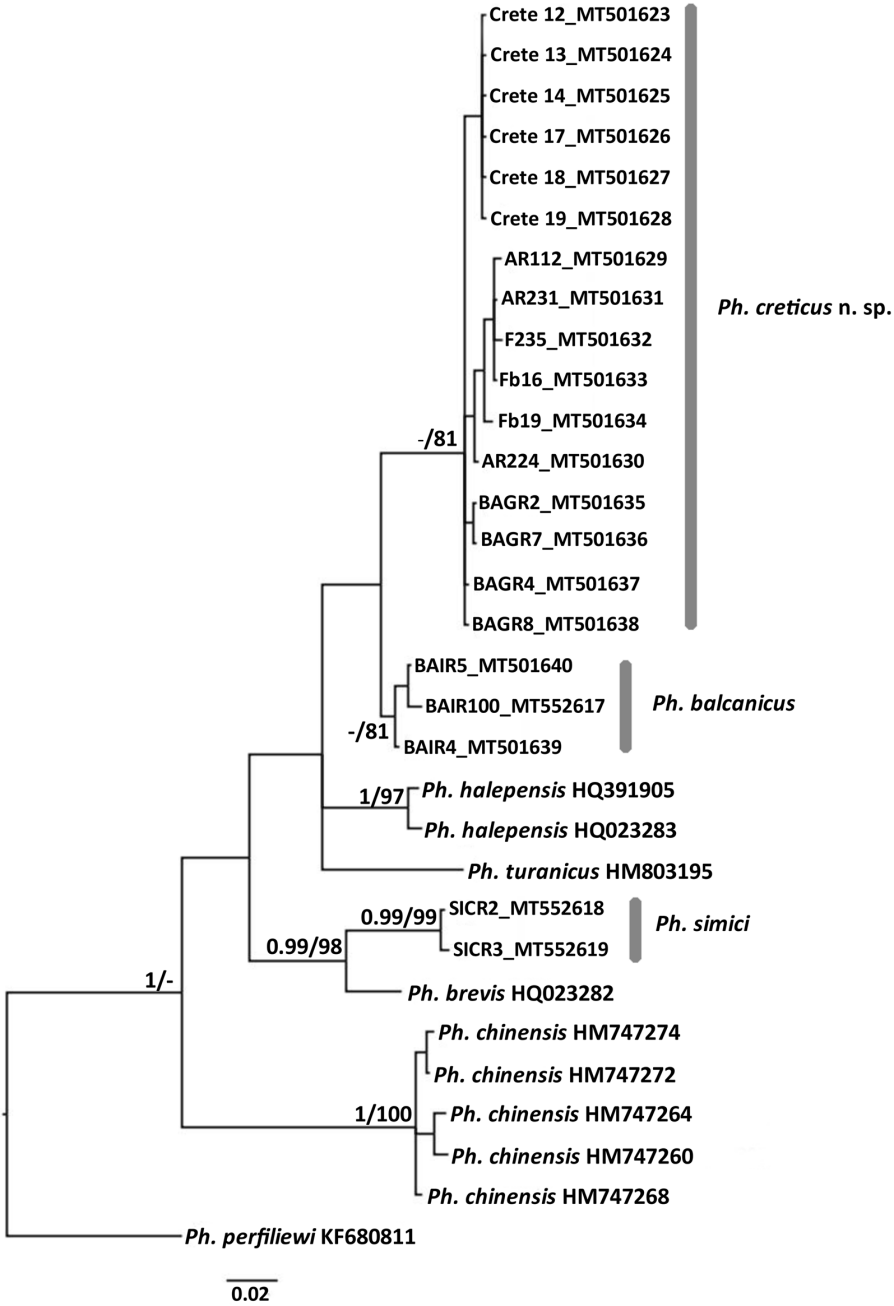
Bayesian inference phylogenetic tree, including posterior probabilities computed in the BI analysis (values > 0.95 are shown) and bootstrap values computed in the ML analysis (values > 70 are shown)

The best-fit nucleotide substitution model for *cytb* was Hasegawa-Kishino-Yano (HKY) + I for all codon positions and for both MrBayes and PhyML. Both analyses led to phylogenetic trees with similar topologies and hence, only the consensus MrBayes tree including posterior probabilities and bootstrap values is presented here (Fig. 3). All species used in these analyses formed monophyletic clades and specifically, the specimens of *Ph. creticus* n. sp. formed a well-supported monophyletic clade. *Phlebotomus creticus* n. sp. appears to be more closely related to *Ph. balcanicus* than the other species included in the analyses.

### MALDI-TOF protein profiling

**Fig. 4:**
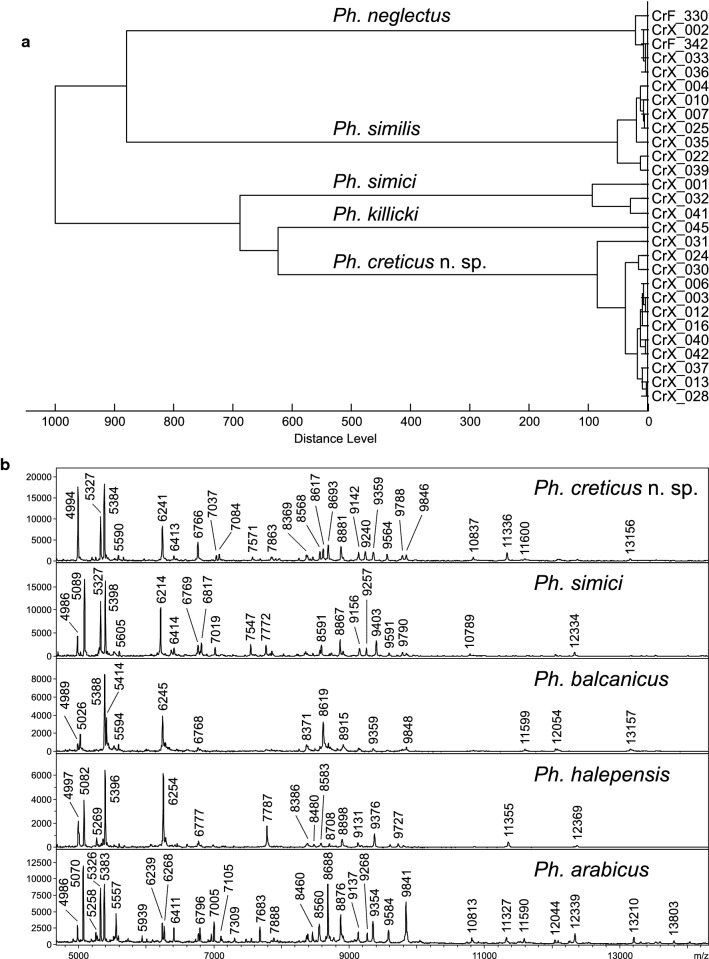
MALDI-TOF mass spectrometry of *Phlebotomus creticus* n. sp. **a** Dendrogram obtained by cluster analysis of MALDI-TOF MS protein profiles of 28 sand fly specimens collected in Crete. Distances are displayed in relative units. **b** Comparison of protein spectra of *Ph. creticus* n. sp. with four species of the subgenus *Adlerius*, zoomed mass range 4–15 kDa

In total, 28 specimens of five *Phlebotomus* species from Xerokampos (*n* = 26) and Fodele (*n* = 2) were analysed: *Ph. creticus* n. sp. (*n* = 12), *Ph. killicki* (*n* = 1), *Ph. neglectus* (*n* = 5), *Ph. similis* (*n* = 7) and *Ph. simici* (*n* = 3). Reproducible protein spectra with a high number of intense signals within the mass range of 4–25 kDa were generated for all analysed specimens. These protein profiles were species-specific and showed species-unique peaks that allowed reliable and conclusive differentiation of the analyzed specimens. Protein spectra of the specimens identified based on morphology as belonging to four known species were similar to the corresponding reference spectra of the respective species. Protein spectra of all *Ph. creticus* n. sp. specimens were identical and differed substantially from the spectra of other species as shown by a hierarchical cluster analysis (Fig. 4a). Moreover, they also showed a number of specific peaks, not shared by protein spectra of four *Adlerius* species in the reference database (Fig. 4b).

**Family Psychodidae Newman, 1834**

**Genus** ***Phlebotomus*** **Rondani & Berté, 1840**

***Phlebotomus creticus*** **Antoniou, Depaquit & Dvorak n. sp.**

***Type-locality***: Xerokampos (35° 3′ 29.37′′ N, 26° 14′ 27.14′′ E; altitude: 19 m above sea level), Greece

***Other localities***: Fodele (35° 22′ 53.93′′ N, 24° 57′ 28.27′′ E; altitude: 48 m above sea level); Agia Roumeli (35° 13′ 24.77′′ N, 23° 56′ 7.53′′ E; altitude: 14 m above sea level); Toplou Monastery (35° 13′ 16.69′′ N, 26° 12′ 51.99′′ E; altitude: 159 m above sea level), Greece.

***Type-material***: The holotype male (accession no. ED10723) and five paratypes (3 males and 2 females, accession nos. ED10724, ED10725, ED10726 for male paratypes, accession nos. ED10727, ED10728 for female paratypes) have been deposited at the Laboratory of Entomology of the Muséum National dʼHistoire Naturelle, Paris, France. Two paratypes (1 male and 1 female) have been deposited at the Museum of Natural History of London, UK, under the accession numbers NHMUK-ENT-2020-42. Two paratypes (1 male and 1 female) have been deposited at the Museum of Natural History of Heraklion, Crete, Greece, under the accession numbers NHMC.85.4.17830.1 (male paratype) and NHMC.85.4.17830.2 (females paratype).

***Representative DNA sequences***: GenBank accession numbers MT501628-MT501638.

***ZooBank registration***: To comply with the regulations set out in Article 8.5 of the amended 2012 version of the *International Code of Zoological Nomenclature* (ICZN) [33], details of the new species have been submitted to ZooBank. The Life Science Identifier (LSID) of the article is urn:lsid:zoobank.org:pub:D2A4798C-A59B-4851-819F-DA238621A474. The LSID for the new name *Phlebotomus creticus* is urn:lsid:zoobank.org:act:218CBCD4-875D-48E1-B2B8-749D53E817DA.

***Etymology***: The new species is named after the island where it has been discovered.

### Description

**Fig. 5:**
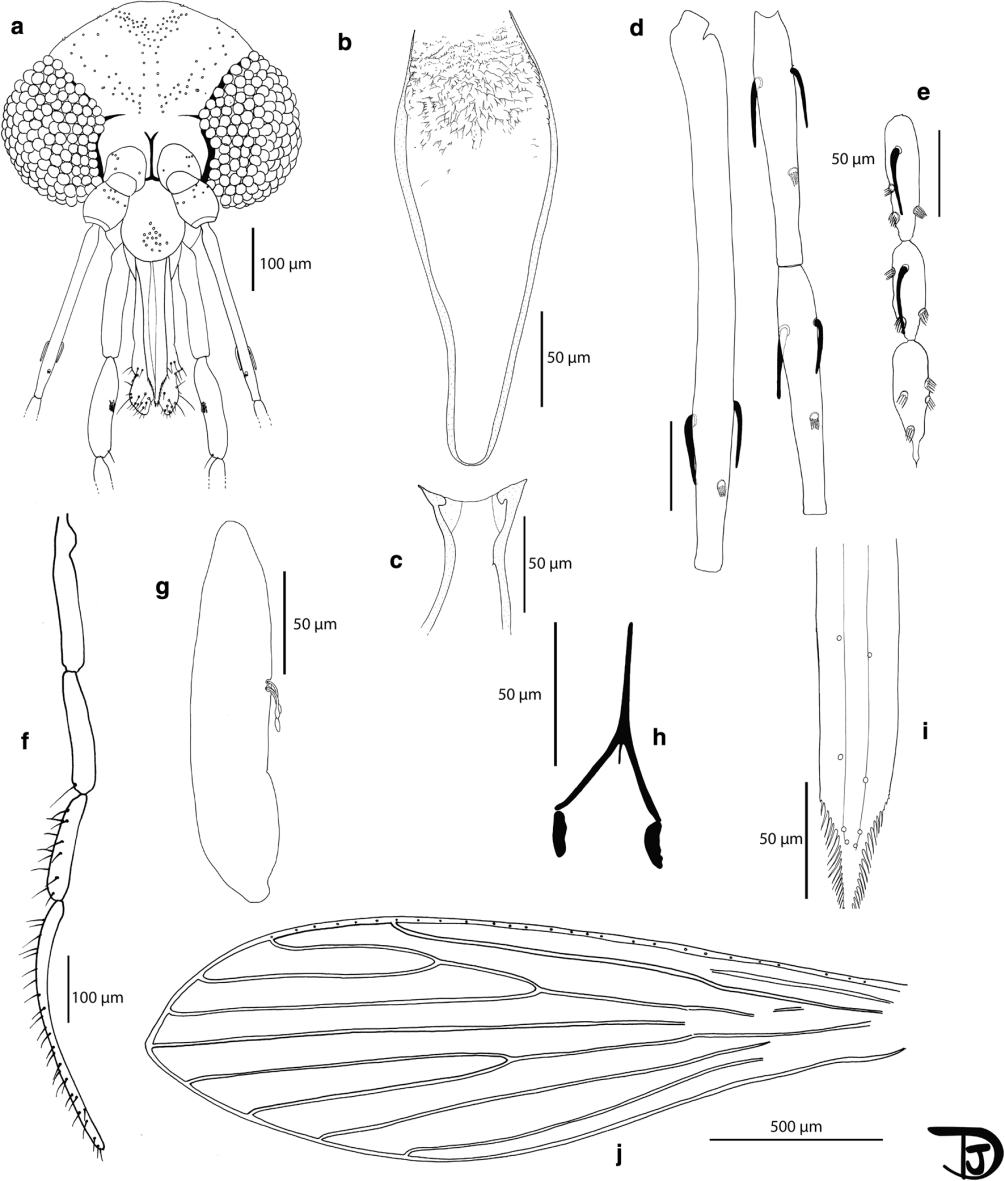
*Phlebotomus creticus* n. sp. male. **a** Head. **b** Pharynx. **c** Cibarium. **d** Flagellomeres 1, 2 and 3. **e** Flagellomeres 12, 13 and 14. **f** Palp. **g** Third palpal article. **h** Labial furca. **i** Labrum. **j** Wing

**Fig. 6:**
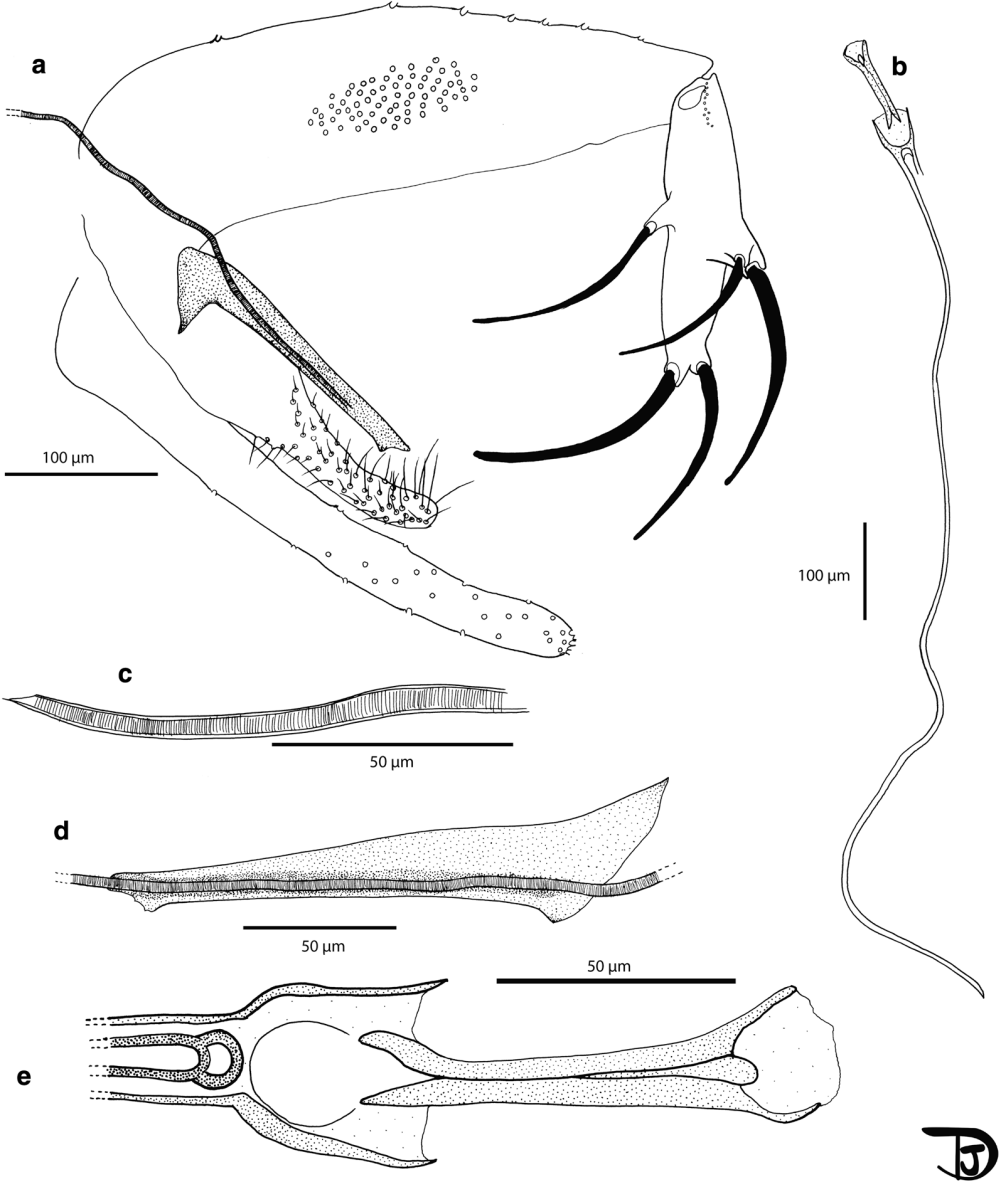
*Phlebotomus creticus* n. sp. male. **a** Genitalia. **b** Sperm pump and aedeagal ducts. **c** Top of the aedeagal ducts. **d** Parameral sheath. **e** Sperm pump

***Male*** [Based on 54 specimens. Counts and measurements indicated in the description are those of the holotype. More measurements for males are available in Table 6; Figs. 5, 6]. Total length 3.9 mm. *Head*. Occiput with 2 lines of well individualized setae. Clypeus 171 µm long, 92 µm wide, with *c.*19 setae randomly distributed, targeting center of clypeus. Eyes 242 µm high with *c.*120 facets. Interantennal suture incomplete. Interocular suture not reaching the interantennal suture. Flagellomeres: f1 (349 µm) longer than f2 (148 µm) + f3 (149 µm). Internal and external ascoids implanted more or less at the same level on f1 to f3. Ascoids not reaching the next articulation. Ascoidal formula: 2/f1-f3 1/f4-f13 (= 2/III-V 1/VI-XV). One distal papilla on flagellomeres f1, f2, f3, three sensillae on f12 and f13, five on f14. Palpi p1: 49 µm long, p2: 173 µm, p3: 192 µm, p4: 160 µm, p5: 400 µm. Palpal formula: 1, 4, 2, 3, 5. About 15 Newstead’s sensilla present on p3 only; no sensilla on other palpal articles. One distal spiniform seta on p3, 12 setae on p4 and 26 setae on p5. Labrum-epipharynx 288 µm long carrying long teeth at its top. Hypopharynx with 20 long apical teeth. Labial suture closed, narrow, in furca. Cibarium without teeth nor sclerotized area (= pigment patch) or sclerotized arch. Pharynx with an armature consisting of long teeth directed laterally or towards center. Cervix with 3 lateral cervical sensillae and 2 median sensillae on each side. *Thorax.* Sclerites pale coloured. One post-alar seta present on the mesonotum. Paratergital seta absent. A group of four proepimeral setae. Upper anepisiternal seta, lower anapisternal seta, anepimeral seta, metaepisternal seta and metaepimeral seta absent. Setae present on the anterior region of the katepisternum. Metafurca mounted in lateral view on all specimens. Vertical arms long, probably separate; horizontal arms long. *Wings*. Length: 2370 μm; width: 715 μm; r5: 1537 µm; α (r2): 475 μm; β (r2+3): 309 μm; γ (r2+3+4): 373 μm; δ: 120 μm; π: 90 μm; ε (r3): 692 μm; θ (r4): 1091 μm; width/γ: 1.92. *Legs*. Anterior leg: coxa: 337 µm; femur: 855 µm; tibia: 1021 µm; tarsomere i: 620 µm; sum of tii, tiii, tiv, tv: 770 µm. Median leg: coxa: 353 µm; femur: 870 µm; tibia: 1218 µm; tarsomere i: 704 µm; sum of tii, tiii, tiv, tv: 810 µm. Posterior leg: coxa: 394 µm; femur: 1005 µm; tibia: 1558 µm; tarsomere i: 887 µm; sum of tii, tiii, tiv, tv: 944 µm. Spines on the metafemur absent. Metatarsomere iii with 4 verticils including broad and thin spines. *Abdomen*. Setae randomly implantated on tergites II to V. Two papillae with hair present on tergites III to VI. Gonocoxite 441 μm long. Sclerotized band in the ventral margin abent; process absent; a median cluster of 71 setae on the internal side present. Gonostyle 204 µm long, with 5 spines: 2 distal spines implanted at the same level; 3 median spines: 1 ventral implanted on narrow tubercle and 2 dorsal implanted on wide tubercle. Parameres 462 µm long, simple, rounded at apex, with setae occupying inner face of distal half. Parameral sheath straight, 203 µm long, with very shallow subterminal tubercle 20 µm to the top. Aedeagal ducts 927 μm long, isodiametric and pointed at their tops. Sperm pump 145 μm long. Ejaculatory apodeme 119 µm long. Aedeagal ducts/sperm pump ratio: 6.39. Epandrial lobes slightly longer than gonocoxites, length 468 μm.

**Fig. 7:**
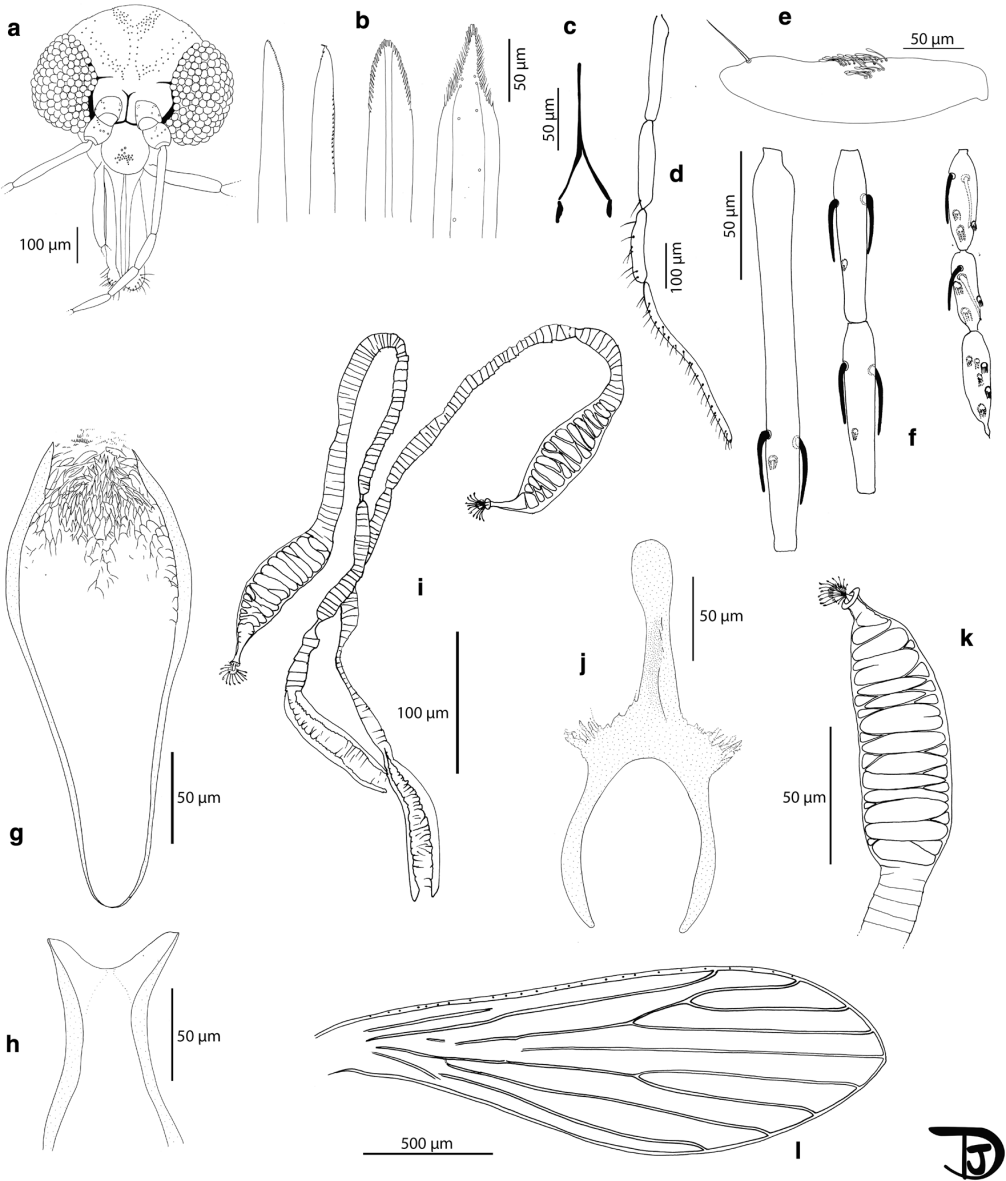
*Phlebotomus creticus* n. sp. female. **a** Head. **b** Mouth parts (mandible, maxilla, hypopharynx and labrum, respectively). **c** labial furca. **d** Palp. **e** Third palpal article. **f** Flagellomeres 1, 2, 3, 12, 13 and 14; **G** pharynx. **h** Cibarium. **i** Spermathecae. **j** Genital fork. **k** Body of the spermathecae. **l** Wing

***Female*** [Based on 11 specimens. Counts and measurements indicated in the following description are those of the paratype labelled Crete IT8 with some exceptions which are indicated. More measurements for females available in Table 7; Fig. 7]. Total length of the paratype Crete IT14: 3.6 mm long. *Head.* Occiput with two narrow lines of well individualized setae. Clypeus 135 µm long, 103 µm wide, with 20 setae randomly distributed, targeting the center of the anterior part of the clypeus. Eyes 193 µm high with about 90 facets. Interantennal suture incomplete. Interocular suture not reaching the interantennal one. Flagellomeres: f1 (208 µm) longer than f2 (98 µm) +f3 (100 µm). Internal and external ascoids implanted more or less at the same level on f1 to f3. Ascoids not reaching the next articulation. Ascoidal formula: 2/f1-f13 (= 2/III-XV). One papilla on flagellomeres f1, f2, f3, three sensillae on f12 and f13, five on f14. Palpi p1: 40 µm long, p2: 153 µm, p3: 160 µm, p4: 130 µm, p5: 315 µm. Palpal formula: 1, 4, 2, 3, 5. Presence of about 15 Newstead’s sensilla on p3. Absence of Newstead’s sensilla on the other palpal articles. One distal spiniform setae on p3,7 on p4 and 31 on p5. Labrum-Epipharynx 268 μm long. f1/E = 0.78. Hypopharynx with about 15 distal long teeth on each side. Maxillary lacinia exhibiting 4 external and 20 internal teeth. Labial suture closed, narrow, in furca. Cibarium without teeth nor sclerotized area (= pigment patch) or sclerotised arch. Pharynx with a triangular armature consisting of elongated teeth directed towards the center. Cervix with two lateral cervical sensillae and two median ones on each side. *Thorax.* Pale coloured sclerites. Presence of one post-alar seta on the mesonotum. Absence of paratergital seta. A group of six proepimeral setae. Absence of upper anepisiternal seta. Absence of lower anapisternal seta. Absence of anepimeral seta. Absence of metaepisternal seta. Absence of metaepimeral seta. Presence of setae in the anterior region of the katepisternum. Metafurca mounted in lateral view on all specimens. Long vertical arms probably separate. Long horizontal arms.738. *Wings.* Length = 2173 μm, width = 578 μm, r5 = 1424 µm, α (r2) = 431 μm, β (r2+3) = 225 μm, γ (r2+3+4) = 398 μm, δ = 101 μm, π = 61 μm. ε (r3) = 612 μm, θ (r4) = 944 μm. Width / γ = 1.70. Anterior leg: coxa = 273 µm; femur = 811 µm; tibia = 971 µm; tarsomere i = 595 µm; sum of tii, tiii, tiv, tv = 728 µm. Median leg: coxa = 328 µm; femur = 817 µm; tibia = 1150 µm; tarsomere i = 686 µm; sum of tii, tiii, tiv, tv = 775 µm. Posterior leg: coxa = 383 µm; femur = 890 µm; tibia = 1522 µm; tarsomere i = 848 µm; sum of tii, tiii, tiv, tv = 876 µm. Absence of spines on the metafemur. Metatarsomere iii with a distal verticil and a median one, including broad and thin spines. *Abdomen.* Setae randomly implantated on tergites II to V. Presence of papillae on tergites III to VII. *Genitalia.* Presence of about 8 + 1 setae on tergite VIII. Lack of protuberance on tergite IX. Spermathecae incompletely segmented. Basal part of the ducts wide with thick walls. Those of the paratype Crete IT8 have been collapsed during the mounting. The measurements indicated are those of the paratype crete7 mounted in Marc-André to be observed, measured and drawn before final process and mounting. Length of the ducts: 600 µm (including 100 µm of the wide basal part and 500 µm of the narrow ducts); length of the body: 100 µm. Genital fork 192 µm long. Cerci rounded at their top, 178 µm long. No seta observed on the sternite X.

### Differential diagnosis

In males, number of ascoids usually 2/f1-f3, 1/f4-f13, a range of 54–85 setae on the gonocoxal internal tuft equally distributed between the proximal and distal halves of the gonocoxite.

### Microhabitat preferences of *Ph. creticus* n. sp.

At Fodele, *Ph. creticus* n. sp. was captured in four CDC light traps placed near or in cave entrances, facing southeast and south. The vegetation around was rich. Mainly chicken, rats and lizards dwell around the traps. No specimens of *Ph. creticus* n. sp. were collected by sticky traps. At Xerokampos, the species was captured in two traps placed at the entrance of two shallow caves facing southeast, at 200 m distance from the shore. The vegetation around the caves was composed of phrygana, biotope typical for island of Crete. At Agia Roumeli the species was captured in two traps placed at the entrance of two limestone rock caves, one facing east and the other facing south, in a separate beach located west of Agia Roumeli, just a few meters from the sea. The vegetation above the caves was composed from sparse phrygana. In Toplou Monastery, it was trapped in caves along the wall just before the Monastery.

## Discussion

The island of Crete is an important and long-time active region of leishmaniases transmission and still provides ample numbers of human cases annually. As sand flies are the only proven vectors of these diseases in the Mediterranean basin, there is a need for sustained monitoring of the sand fly fauna and its role in *Leishmania* transmission. While studied for a long time, there are surprising new findings regarding the presence of sand fly species as documented by a recent description of *Phlebotomus* (*Transphlebotomus*) *killicki* Dvorak, Votypka & Volf, 2015 in the localities at the southern coast of the island [49]. The results of the presented study reveal that there is still more to discover.

Published data as well as new results from the present sampling (2014–2019) show that the most widespread and abundant species in Crete is *Ph. neglectus*, accounting for more than 60% of the recorded sand fly specimens. It is a proven vector of *L. infantum* in Greece, several other Balkan countries and the western part of Turkey [1]. Its abundance in all studied areas of Crete apparently contributes to the geographical distribution of VL cases throughout the island and as other species of the subgenus *Larroussius* are markedly scarce, we may conclude that *Ph. neglectus* remains a sole vector of the disease. The second most common and abundant species on the island is *Phlebotomus similis*. It is morphologically similar and phylogenetically closely related to *Ph. sergenti* [51, 52] and is regarded as a suspected vector of *L. tropica* in regions where *Ph. sergenti* is not present, including Crete [2]. The fact that *Ph. similis* was found in all foci of human CL further fosters the considerations of this species as a yet unproven vector of *L. tropica* in the island. *Phlebotomus papatasi* is a proven vector of *L. major* in the Middle East and Northern Africa and it is known to be responsible for the phlebovirus infections in the Old World [53, 54] including Crete [55]. However, *L. major*, the causative agent of zoonotic cutaneous leishmaniasis, does not circulate in Crete, probably due to absence of suitable reservoir species (gerbils). Vectorial competence of *Sergentomyia* spp. to mammalian diseases is still controversial and experimental studies that would conclusively test it are scarce [56] despite growing circumstantial evidence that suggest incrimination of some species in the transmission of human leishmaniasis and phleboviruses [57]. Of two *Sergentomyia* species previously recorded from Crete, only *Se. minuta* was found in this study. Bloodmeal analyses of population in southern Portugal recently demonstrated that this widely distributed Mediterranean species may be at least partially anthropophilic and the detection of *Leishmania* DNA in specimens from the same area emphasizes the need of further studies about the role of *Sergentomyia* species in leishmaniases transmission cycles [58].

*Adlerius* specimens collected at different sites throughout the island during 2014–2019 did not meet the criteria of any known species and exhibited unique morphological characters. Moreover, obtained sequences of *cytb*, when compared with sequences of species within the subgenus *Adlerius* available in public databases, were substantially different. That led us to the conclusion that the collected specimens represent a new species. The identity of this new species is based on a detailed morphological analysis of decisive characters that is further supported by sequencing of *cytb* gene, a widely used genetic marker, and for the first time in a description of a new sand fly species, also by comparison of species-specific protein spectra acquired by MALDI-TOF mass spectrometry.

According to Artemiev [10], the subgenus *Adlerius* includes 17 described and two undescribed species. While the females mostly appear to be undistinguishable, the main characters to identify the males of this subgenus are the antennal formula, used in all available identification keys [10, 11, 59] and the number of setae of the internal tuft of the gonocoxite as well as the position of this tuft on the gonocoxite.

Considering that the number of ascoids in *Ph. creticus* n. sp. was usually 2/f1-f3, 1/f4-f13, the most similar species are *Ph. angustus* Artemiev, 1978, *Ph. balcanicus*, *Ph. comatus* Artemiev, 1978, *Ph. kyreniae* Theodor, 1958, *Ph. salangensis* Artemiev, 1978 and *Ph. zulfagarensis* Artemiev, 1978 [10]. However, this parameter varied not only in *Ph. creticus* n. sp., its infraspecific variation is known also in other sand fly species. Therefore, the antennal formula may not be alone a useful parameter for species identification of *Adlerius* sand flies.

*Phlebotomus creticus* n. sp. exhibits a range of 54–85 setae on the gonocoxal internal tuft (mean: 69). This count excludes the identification of *Ph. balcanicus* (92–130 setae after Artemiev [10] and more than 100 in the original description [reference number]), *Ph. comatus* (126–220 setae) and *Ph. kyreniae* (30–40 setae). There is an overlap related to this number of setae for *Ph. creticus* n. sp. and *Ph. angustus* (35–69 setae), *Ph. salangensis* (40–85 setae) and *Ph. zulfagarensis* (66–72 setae). However, these three species exhibit group of setae which is completely or at 90% in the proximal half of the gonocoxite [10] whereas that of *Ph. creticus* n. sp. is equally distributed between the proximal and distal halves of the gonocoxite (Table 6, Fig. 1). Moreover, all three species are known to be distributed in Asia, in regions very distant from Crete. In contrast, in *Ph. balcanicus* the hair group is located mainly on the basal half of the coxite [12]. Consequently, regarding the typological systematics, there is substantial evidence to consider *Ph. creticus* as a distinct species.

*Phlebotomus creticus* n. sp. was recorded at different localities at both northern and southern coast of Crete divided by a mountain range of a considerable height, which further supports a long presence rather than a recent introduction. However, it is probably not a common species. *Phlebotomus creticus* n. sp. was not caught on sticky traps placed in the openings of holes on natural or manmade walls and ground. We assume that it preferentially rests inside shallow caves in limestone rocks. In such specific biotopes it could be a dominant species as demonstrated in Xerokampos. Its feeding preferences should be investigated, although wildlife hosts may be expected (mice, birds and possibly lizards). Beside this newly described species, *Ph. simici* of the same subgenus that had been reported from Crete in the past, was still recorded in this study, occurring even sympatrically at the type-locality Xerokampos. We may speculate that in previous entomological surveys, some of the specimens identified as *Ph. simici* or unidentified *Adlerius* sp. may be attributed to the newly described *Ph. creticus* n. sp.

The results of the molecular analyses support the description of a new species. Phylogenetic analyses of the *cytochrome b* gene, a mitochondrial marker widely used in phylogenetic studies of many insect groups including sand flies, strongly grouped all analyzed specimens of *Ph. creticus* n. sp. in a distinct monophyletic clade. This genetic marker was chosen as it provides the best coverage of the species within the subgenus *Adlerius*. Unfortunately, no sequences of any genetic marker are so far available for some species of the subgenus, including the three species with overlapping numbers of setae on the gonocoxal internal tuft. Genetic distance values obtained for *Ph. creticus* n. sp. and other compared *Adlerius* species are comparable with the distances recorded previously for other sand fly species, as shown by studies of morphologically- as well as genetically distinct species of the subgenera *Larroussius* and *Phlebotomus* [60] or *Madaphlebotomus* [9]. Moreover, MALDI-TOF protein profiling demonstrated that all processed specimens of *Ph. creticus* n. sp. produced unique, reproducible and species-specific profiles that clearly differentiate them from other species outside and within the subgenus *Adlerius*. This method of mass spectrometry has recently become a popular tool for species identification of various organisms including arthropod vectors as it is simple, rapid and cost-effective [61]. Here, we demonstrated for the first time that it can also successfully complement traditional morphological approach and established DNA-based molecular taxonomy in the process of revealing yet unrecognized sand fly species.

Our recent findings urge the need for a revision of the subgenus *Adlerius* using both morphological and molecular approaches. Species boundaries are not well defined and the vicariance of this group probably occurred recently as for other groups of phlebotomine sand flies such as *Phlebotomus*, *Larroussius* or *Paraphlebotomus* [16, 18, 62].

## Conclusions

In this study we present a review of sand fly species recorded in the past and at present in Crete, an island with ongoing transmission of two *Leishmania* species due to the presence of competent sand fly vectors. The importance of this research is highlighted by the geographical position of the island and the current possibility of accidental introduction of more *Leishmania* species due to human migration and other activities. According to our findings, 10 *Phlebotomus* spp. and 2 *Sergentomyia* spp. were recorded, with the most common and abundant species being *Ph. neglectus*. We may assume that the findings of *Ph. mascittii* reported prior to the description of *Ph. killicki* may be attributed to the latter species. We identify and describe a new species *Phlebotomus* (*Adlerius*) *creticus* n. sp. from various localities in Crete. Its identification is based on morphological characters of the male genitalia that particularly differentiate it from related species of the subgenus *Adlerius*. The identity of the newly described species was confirmed by two molecular approaches (MALDI-TOF protein profiling and *cytb* sequence analysis). As there is no data on the vectorial competence and capacity of this new species, its potential role in the autochtonous transmission cycles of *Leishmania* shall be further studied.

## Data Availability

The datasets supporting the conclusions of this article are included within the article and its additional files. The holotype and paratypes of a newly described species *Phlebotomus creticus* n. sp.were deposited in three repositories as described above. The newly generated sequences were deposited in the GenBank database under the accession numbers MT501623-MT501638.

## Abbreviations

BI: Bayesian inference
BIC: Bayesian information criterion
CL: Cutaneous leishmaniasis
*cytb*: *Cytochrome b* gene
MALDI-TOF MS: Matrix-assisted laser desorption/ionization time-o-flight mass spectrometry
ML: Maximum likelihood
PCR: Polymerase chain reaction
VL: Visceral leishmaniasis
